# The Relations of Medicine to Modern Unbelief

**Published:** 1877-04-20

**Authors:** 


The Relations of Medicine to Modern Unbelief: A Valedictory Address by Richard O. Cowling, A.M., M.D., delivered at the Thirty-ninth Commencement of the University of Louisville.

